# A Multipopulation Coevolutionary Strategy for Multiobjective Immune Algorithm

**DOI:** 10.1155/2014/539128

**Published:** 2014-02-12

**Authors:** Jiao Shi, Maoguo Gong, Wenping Ma, Licheng Jiao

**Affiliations:** Key Laboratory of Intelligent Perception and Image Understanding of Ministry of Education of China, Xidian University, Xi'an 710071, China

## Abstract

How to maintain the population diversity is an important issue in designing a multiobjective evolutionary algorithm. This paper presents an enhanced nondominated neighbor-based immune algorithm in which a multipopulation coevolutionary strategy is introduced for improving the population diversity. In the proposed algorithm, subpopulations evolve independently; thus the unique characteristics of each subpopulation can be effectively maintained, and the diversity of the entire population is effectively increased. Besides, the dynamic information of multiple subpopulations is obtained with the help of the designed cooperation operator which reflects a mutually beneficial relationship among subpopulations. Subpopulations gain the opportunity to exchange information, thereby expanding the search range of the entire population. Subpopulations make use of the reference experience from each other, thereby improving the efficiency of evolutionary search. Compared with several state-of-the-art multiobjective evolutionary algorithms on well-known and frequently used multiobjective and many-objective problems, the proposed algorithm achieves comparable results in terms of convergence, diversity metrics, and running time on most test problems.

## 1. Introduction 

Optimization problems widely exist in real life, especially in engineering applications [1–4]. The optimization problem with only one objective is called single-objective optimization problem. The optimization problem with more than one objective to be simultaneously solved is called multiobjective optimization problem (MOP). In practical optimization applications, there is a great demand for optimizing multiple objectives simultaneously. As a heuristic searching method, evolutionary computation has already been successfully used in the field of MOPs and gradually develops into a hot research direction, named evolutionary multiobjective optimization (EMO) [5–8]. The search technique based on population is proved to have a good ability of global searching and can find a set of solutions in one-shot operation. Thus, evolutionary computation achieves comparable results in solving nonconvex, nonlinear, discontinuous and differentiable problems and overcomes the deficiency of traditional mathematical programming [9–13].

The first study on multiobjective evolutionary algorithm (MOEA) is probably the vector evaluated genetic algorithm (VEGA) [14]. Since then, MOEAs have obtained increasing attention, and the amount of literatures about MOEAs has increased in which many MOEAs were designed one after another, such as multiobjective genetic algorithm (MOGA) [15], niched Pareto genetic algorithm (NPGA) [16], and nondominated sorting genetic algorithm (NSGA) [17]. These algorithms are regarded as the typical representatives of the first generation of MOEAs which are characterized by using Pareto ranking-based selection and fitness sharing strategy [18]. The second generation of MOEAs are characterized by using elite strategy, including strength Pareto evolutionary algorithm (SPEA) [19], improved version of SPEA (SPEA2) [20], Pareto envelop-based selection algorithm (PESA) [21], niched Pareto genetic algorithm 2 (NPGA2) [22], and nondominated sorting genetic algorithm II (NSGA-II) [23]. Recently, researches on evolutionary multiobjective optimization present new characteristics. The concepts of simulated annealing [24], particle swarm [25], quantum [26, 27], and messiness [28] were proposed and introduced into the framework of evolutionary algorithms. At the same time, many new-type evolutionary mechanisms were introduced, including regularity-model-based multiobjective estimation of distribution algorithm (RM-MEDA) [29] and multiobjective evolutionary algorithm based on decomposition (MOEA/D) [30].

The concept of artificial immune systems (AIS) was first put forward in 1996. Since then, AIS have stepped into a high-speed development period and become one of the hot topics in the field of artificial intelligence. AIS that get the inspiration from biological immune systems attempt to develop computational tools for solving science and engineering problems. Some AIS-based multiobjective optimization algorithms have been proposed [31–34], including immune optimization algorithm for constrained nonlinear multiobjective optimization problems [34], a hybrid immune multiobjective optimization algorithm [31], and chaos-based multiobjective immune algorithm [32]. Recently, a multiobjective immune algorithm with nondominated neighbor-based selection (NNIA) was proposed by Gong et al. [35]. From the comparison with representative algorithms, it is apparent that NNIA is an effective immune multiobjective algorithm in solving MOPs. Although the employment of elite strategy improves the convergence rate of MOEA, it leads to the loss of population diversity as well. Like the common problem existing in evolutionary algorithms, premature convergence also haunts NNIA. It may be trapped into local optimal solution, thus the population diversity of NNIA needs to be improved.

An enhanced version of nondominated neighbor-based immune algorithm with a multipopulation coevolutionary strategy is proposed for improving the population diversity. Subpopulations employ evolutionary operations independently; thus the unique characteristics of each subpopulation can be effectively maintained. During evolutionary search, information exchanges among subpopulations thus expanding the search range of the entire population. As a matter of fact, most of the evolutionary algorithms employ regular operations throughout the whole evolutionary process, and few of them take advantage of online discovered information. The adaptive operator which dynamically applies evolution operations to subpopulations based on the online discovered information is designed. Therefore, evolutionary search becomes more directional and purposeful and the unnecessary waste of computational cost is reduced.

The remainder of this paper is organized as follows. The problem statement is described in Section 2.  Section 3 presents the proposed algorithm in detail.  Section 4 presents experimental results, ZDT problems, DTLZ problems, and some extensional problems are adopt, and the sensitivity of the introduced parameter, the scalability of the proposed algorithm, and the comparison of running time are also investigated in this section. Finally, we outline the conclusions of this paper.

## 2. Problem Statement

The mathematical description of multiobjective optimization problems can be expressed as follows [36, 37]:

(1) min⁡ y=F(x)=(f1(x),f2(x),…,fm(x))T,s.t.: gi(x)≤0, i=1,2,…,q,    hj(x)=0, j=1,2,…,p,

where *x* = (*x*_1_, *x*_2_,…, *x*_*n*_) ∈ *X* ⊂ *R* ^*n*^ is a decision variable vector, *X* is the decision space, *F*(*x*) is the set of objective functions to be optimized simultaneously, *g*_*i*_(*x*) defines the inequality constraint, and *h*_*j*_(*x*) defines the equality constraint. Based on these mathematical descriptions, several important definitions of multiobjective optimization problems are given as follows.

**Definition 1 (feasible solution and feasible solution set)**

For a certain decision variable vector *x* ∈ *X*, if it satisfies both equality constraints and inequality constraints, then *x* can be called a feasible solution. The feasible solution set is made up of all the feasible solutions, which can be denoted as *X*_*f*_, where *X*_*f*_⊆*X*.

**Definition 2 (Pareto domination)**

For any two feasible solutions, if and only if they satisfy condition (2), it is called that *x*_*a*_ dominates *x*_*b*_, which can be denoted as *x*_*a*_≻*x*_*b*_. Consider the following:

(2) ∀i=1,2,…,m, fi(xa)≤fi(xb)∧∃j=1,2,…,m,fj(xa)≤fj(xb).

**Definition 3 (Pareto-optimal solution and Pareto-optimal set)**

For a certain feasible solution x*, if and only if it satisfies the condition: ¬∃*x* ∈ *X*_*f*_ : *x*≻x*, then x* can be regarded as the Pareto-optimal solution. The Pareto-optimal set is made up of all the Pareto-optimal solutions in the decision space, which can be denoted as

(3) $$\begin{array}{c} P^{*}=\left\{x^{*}\;|\;\neg \exists x\in X_{f}:x≻x^{*}\right\}. \end{array}$$

**Definition 4 (Pareto-optimal front)**

The corresponding image of the Pareto-optimal set in the objective space is called the Pareto-optimal front, which can be denoted as

(4) $$\begin{array}{c} PF=\left\{F\left(x^{*}\right)={\left(f_{1}\left(x^{*}\right),f_{2}\left(x^{*}\right),\ldots ,f_{m}\left(x^{*}\right)\right)}^{\text{T}}\;|\;x^{*}\in P^{*}\right\}. \end{array}$$

In solving MOPs, it is expected that the set of nondominated solutions obtained by the proposed algorithm can well approximate the true Pareto-optimal front and the diversity of the solutions can be maximized.

## 3. Multipopulation Coevolution Multiobjective Immune Algorithm

Many new-type evolutionary methods have been introduced into the area of MOEAs. Immune-based algorithm is one of these late-model methods. Artificial immune systems (AIS) get the inspiration from biological immune systems. They have learnable, parallel, and distributed characteristics, therefore possessing an efficient information processing ability. AIS-based algorithms have attracted a lot of attention and have been applied to many complex MOPs, including constrained nonlinear MOPs and dynamic MOPs [38, 39]. Recently, Gong et al. [35] presented a multiobjective immune algorithm with nondominated neighbor-based selection (NNIA), which is one of the representative immune-based multiobjective algorithms.

### 3.1. The Original NNIA

**Figure 1:**
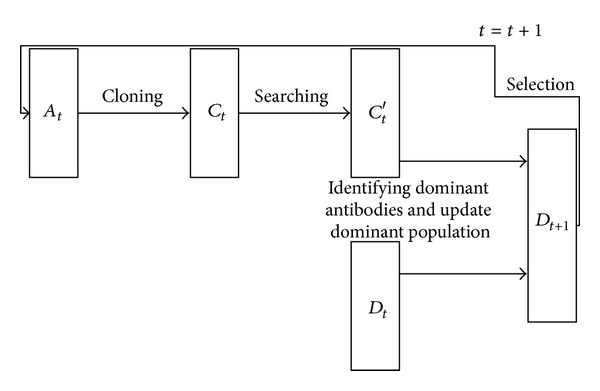
Population evolution of NNIA.

In NNIA, a nondominated neighbor-based selection and a crowding-distance-based proportional cloning were proposed. The fitness of a nondominated individual is assigned according to its crowding distance. The individual with greater crowding distance is reproduced more times and then less-crowded regions will have more chances to be searched, which improves the search ability of NNIA on less-crowded regions. Besides, only a minority of nondominated individuals will be selected to form an active population, and then a series of evolutionary operations are applied to this active population. Therefore, NNIA evolves very fast by performing evolutionary operations on a small-scale active population. The specific framework of population evolution in a single generation at time *t* in NNIA is shown in Figure 1.

From Figure 1, it is easy to observe that the evolutionary search in NNIA is very fast and effective due to its specific framework. Although such efficient mechanism achieves a high evolutionary rate, it introduces errors as well. Due to the efficient mechanism, population diversity is quickly decreased, and the resulting solutions may fall into local optimum, which is not a rare case. Under normal circumstances, the use of the elite strategy in MOEAs will lead to the loss of population diversity. The special evolutionary framework of NNIA further exacerbates this knotty problem.  Table 1 shows the results of NNIA on ZDT2 and ZDT4, where NNIA performs 30 independent runs and the maximum size of the active population is 20. However, NNIA always obtains only one nondominated solution on ZDT2 and ZDT4 during 30 runs. The solutions obtained by NNIA are always trapped into local optimum on ZDT2 and ZDT4, which demonstrates the assertion of the analysis above.

### 3.2. Description of the Proposed Algorithm

In this paper, we present an enhanced multipopulation coevolutionary strategy for nondominated neighbor-based immune algorithm, called CONNIA. Different from the traditional evolution, co-evolution recognizes the simultaneous existence of competition and cooperation among populations, which provides a theoretical basis for maintaining the population diversity.

#### 3.2.1. Adaptive Operator

When it comes to the adaptive operator in the field of MOEAs, it mainly refers to adaptively tuning some parameters, such as population size, crossover probability, and mutation probability. However, the adjustment of evolutionary strategy based on evolutionary conditions is seldom involved. The major contribution of the designed adaptive operator is that each subpopulation adaptively selects corresponding operators during the evolution, which makes the evolution become more purposeful and directional. Therefore, the need for unnecessary computing resource existing in random search is avoided effectively.

After performing a series of evolutionary operations on each subpopulation, a way for measuring the evolutionary condition is to identify the nondominated solutions of each subpopulation. The set coverage metric is employed for measuring the relationship between two subpopulations [40]. If a subpopulation has a higher value of the set coverage metric, it contributes more to the formation of the entire approximated Pareto front. The adaptive operator which consists of two different cases is designed on the basis of measuring the relationship between two subpopulations. Different evolutionary operators are designed for different cases. A threshold is introduced to decide which case is activated. The influence of the threshold on the performance is analyzed in Section 4.3.

**Case 1**

If the difference of the set coverage metric between subpopulations is not obvious, a local search operator would be employed. Two subpopulations perform independently evolutionary operations and search within different solution space for maintaining the diversity of the entire population. Meanwhile, some appropriate perturbations are applied around the obtained nondominated solutions for seeking the possible better solutions and reducing the probability of getting into local optimum.

**Case 2**

If the difference of the coverage metric between subpopulations is obvious, a cooperation operator would be employed. The information exchanges among subpopulations, which reflects a mutually beneficial relationship between two subpopulations. The subpopulation with lower value of the set coverage metric could make use of the reference experience from another subpopulation to improve its own evolution. Two subpopulations make progress together by means of cooperation to ultimately complete the evolutionary task.

#### 3.2.2. Local Search Operator and Cooperation Operator

We get the inspiration from traditional differential evolution (DE) operator [41] to design the local search operator and the cooperation operator. DE operator uses the differences between the structures of antibodies to guide the antibody variation and make the generated antibody closer to the optimal point.

*Local Search Operator*. Assume that *P*_*t*_ is a population, *D*_*t*_ is the nondominated population of *P*_*t*_, and two individuals (*x*_1_, *x*_2_,…, *x*_*n*_) and (*y*_1_, *y*_2_,…, *y*_*n*_) are randomly selected from *P*_*t*_ and *D*_*t*_, respectively. A new individual (*z*_1_, *z*_2_,…, *z*_*n*_) is generated through the following operation:

(5) $$\begin{array}{c} z_{i}=y_{i}+U\left(-1,1\right)*\left(y_{i}-x_{i}\right), \end{array}$$

where *i* = 1,2,…, *n*, *U*(·, ·) is a uniformly distributed random number. As we know, there may be some better solutions around the obtained Pareto-optimal solutions, particularly in the case that the obtained Pareto-optimal solutions are trapped into local optimum. The designed local search operator inflicts appropriate disturbances around the obtained Pareto-optimal solutions, and then the opportunity of finding some better solutions is increased. After the local search operation, *a*  (*μ* + *λ*) selection strategy is adopted [42]. This elite strategy ensures the effectiveness of the local search operation and accelerates the rate of evolutionary search.

*Cooperation Operator*. Assume that there are two populations *P*_*t*1_ and *P*_*t*2_. *D*_*t*1_ and *D*_*t*2_ are two nondominated populations of *P*_*t*1_ and *P*_*t*2_, respectively. *P*_*t*2_ is better than *P*_*t*1_ in terms of the set coverage metric. Two individuals (*x*_1_, *x*_2_,…, *x*_*n*_) and (*y*_1_, *y*_2_,…, *y*_*n*_) are randomly selected from *P*_*t*1_ and *D*_*t*2_, respectively. A new individual (*z*_1_, *z*_2_,…, *z*_*n*_) is generated through the following operation:

(6) $$\begin{array}{c} z_{i}=y_{i}+U\left(-1,1\right)*\left(y_{i}-x_{i}\right), \end{array}$$

where *i* = 1,2,…, *n*, *U*(·, ·) is a uniformly distributed random number. By applying the cooperation operator, subpopulations gain the opportunity to exchange information, thus expanding the search range of their own. The subpopulation with larger value of the set coverage metric may possess more effective convergence information. In this case, the subpopulation with lower value of the set coverage metric can improve its evolutionary capacity by gaining the experience from the outstanding antibodies in another subpopulation. This directed cooperation operator provides good evolutionary paths towards antibodies, thereby making antibodies evolve faster when compared with the noncooperation strategy.

The designed local search operator and cooperation operator reflect a mutually beneficial relationship between subpopulations. Both operators transmitting information among antibodies within the same generation are combined with traditional evolutionary operators such as crossover and mutation, for transmitting information effectively.

#### 3.2.3. Multipopulation Coevolutionary Nondominated Neighbor-Based Immune Algorithm

The details of the proposed algorithm are described in this part. To be specific, the following parts are designed. (1) As each subpopulation evolves independently, the differences between subpopulations can be well kept. (2) By means of information exchange among subpopulations, the search range of each subpopulation can be effectively expanded. (3) The way of information exchange depends on the gap of the set coverage metric between subpopulations. Such online-decision strategy has an adaptive character, which improves the global search efficiency. The main steps of CONNIA are presented as follows.

**Step 1**

Generate two initial subpopulations*P*_*a*0_ and *P*_*b*0_.

**Step 2**

The nondominated antibodies of the two subpopulations *P*_*at*_ and *P*_*bt*_ form two nondominated populations *D*_*at*_ and *D*_*bt*_, respectively. Then the two nondominated populations are combined together to form the entire nondominated population *T*_*t*_.

**Step 3**

If the terminal condition is satisfied, export *T*_*t*_ as the output. Stop; otherwise, *t* = *t* + 1.

**Step 4**

Select the individuals which have more contributions to the population diversity from *D*_*at*_ and *D*_*bt*_, respectively. Then the selected individuals form two active populations *A*_*at*_ and *A*_*bt*_.

**Step 5**

Two clone populations *C*_*at*_ and *C*_*bt*_ are formed by applying cloning to *A*_*at*_ and *A*_*bt*_, respectively.

**Step 6**

Perform recombination and mutation on *C*_*at*_ and *C*_*bt*_; then obtain two resulting populations *C*_*at*_′ and *C*_*bt*_′.

**Step 7**

If the condition of information exchange is satisfied, perform cooperation operator between *C*_*at*_′ and *C*_*bt*_′. Otherwise, perform guided local search operator on *C*_*at*_′ and *C*_*bt*_′, respectively. Then recalculate the nondominated solutions of *C*_*at*_′ and *C*_*bt*_′, respectively.

**Step 8**

Get subpopulations *P*_*at*_ and *P*_*bt*_ by combining *C*_*at*_′ and *D*_*at*_, *C*_*bt*_′ and *D*_*bt*_, respectively; go to Step 2.

### 3.3. Solution Pruning Based on Crowding Distance

In the proposed algorithm, the crowding distance [23] is used to estimate the density around a solution and the contribution of a solution to the diversity of objective function values. The definition of the crowding distance is described as follows:

(7) $$\begin{array}{c} D\left(x\right)=\sum_{i=1}^{k}\frac{f_{i}\left(x^{\prime }\right)-f_{i}\left(x^{\prime \prime }\right)}{f_{i}^{\max}-f_{i}^{\min}}, \end{array}$$

where *f*_*i*_^max⁡^ and *f*_*i*_^min⁡^ are the maximum and minimum values of the *i*th objective and *k* is the number of objective functions. *f*_*i*_(*x*′) and *f*_*i*_(*x*′′) are the values of the *i*th objective of the top two nearest points to *x*. If *x* is an extreme point, *D*(*x*) = *∞*. Otherwise, the crowding distance of *x* is calculated by (7).

The density around a dominant antibody is estimated by calculating its crowding distance. The larger the crowding distance of a dominant antibody is, the sparser the distribution around it will be, which also means that the contribution of this antibody to the population diversity is relatively greater. When it is required to delete some solutions, the antibody with small crowding distance will be deleted firstly. The traditional way of solution pruning is to calculate the crowding distance of all solutions only once, and then some solutions are deleted based on such one-shot result. However, such mechanism is unreasonable sometimes.

**Figure 2:**
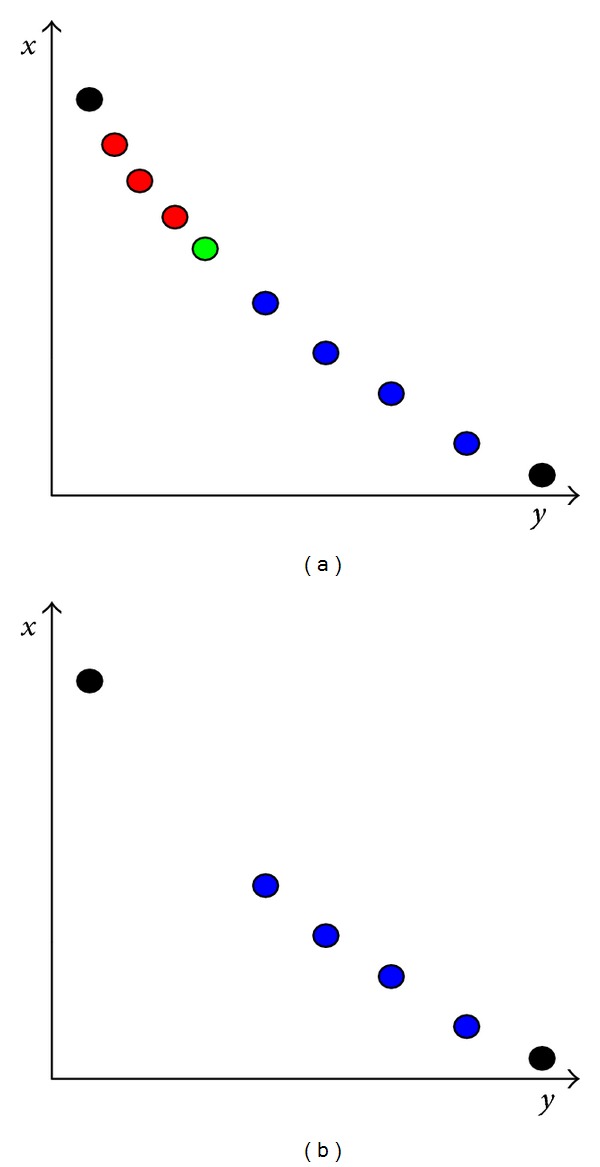
The static method of solution pruning.

After calculating the crowding distance of all points shown in Figure 2(a), it is evident that two black extreme points have the largest crowding distances. In addition to black points, four blue points have larger crowding distances than other points. Points are sorted according to the crowding distance, from black points, blue points, green point, to red points in a decline order. Assume that four points need to be deleted, and then the red and green points are deleted by using the original static method. It is obvious that the points after pruning are not well-distributed as shown in Figure 2(b). It has been mentioned that the dynamic way is more reasonable than the traditional static method [43]. After deleting a point, recalculate the crowding distance of the remaining points and sort them based on the recalculated crowding distance.

### 3.4. Computational Complexity Analysis of CONNIA

Assume that the maximum size of the dominant population is *n*_*d*_, the maximum size of the active population is *n*_*a*_, and the size of the clone population is *n*_*c*_. The time complexity for CONNIA in a single generation without information exchange can be calculated as follows.

The time complexity for identifying nondominated individuals in the population is *O*((*n*_*c*_+*n*_*d*_)^2^); the worst time complexity for dynamic selection is ∑_*i*=*n*_*a*__^*n*_*d*_^ *O*(*i*log⁡⁡*i*); the time complexity for cloning is *O*(*n*_*c*_); the worst time complexity for updating the dominant population is ∑_*i*=*n*_*d*__^*n*_*d*_+*n*_*c*_^ *O*(*i*log⁡⁡*i*); and the time complexity for recombination and mutation is *O*(*n*_*c*_).

Therefore the worst total time complexity is:

(8) $$\begin{array}{c} \sum_{i=n_{a}}^{n_{d}}O\left(i\log i\right)+\sum_{i=n_{d}}^{n_{d}+n_{c}}O\left(i\log i\right)+2O\left(n_{c}\right)+O\left({\left(n_{d}+n_{c}\right)}^{2}\right). \end{array}$$

Owing to the fact that the operational rule of the symbol “*O*” can be simplified, the worst time complexity of one generation without information exchange for CONNIA can be written as: *O*((*n*_*c*_+*n*_*d*_)^2^).

The time complexity for CONNIA in a single generation with information exchange can be calculated as follows.

The time complexity for identifying nondominated individuals in the population is *O*((*n*_*c*_+2*n*_*d*_)^2^); the worst time complexity for dynamic selection is ∑_*i*=*n*_*a*__^*n*_*d*_^ *O*(*i*log⁡⁡*i*); the time complexity for cloning is *O*(*n*_*c*_); the worst time complexity for updating the dominant population is ∑_*i*=2*n*_*d*__^2*n*_*d*_+*n*_*c*_^ *O*(*i*log⁡⁡*i*); and the time complexity for recombination and mutation is *O*(*n*_*c*_).

So the worst total time complexity is:

(9) $$\begin{array}{c} \sum_{i=n_{a}}^{n_{d}}O\left(i\log i\right)+\sum_{i={2n}_{d}}^{{2n}_{d}+n_{c}}O\left(i\log i\right)+2O\left(n_{c}\right)+O\left({\left(2n_{d}+n_{c}\right)}^{2}\right). \end{array}$$

Owing to the fact that the operational rule of the symbol “*O*” can be simplified, the worst time complexity of one generation with information exchange for CONNIA can be written as *O*((*n*_*c*_+2*n*_*d*_)^2^).

In real applications, the key factor to decide whether a technique can be applied is the running time. The further research on the practical running time of the proposed algorithm will be presented in Section 4.9.

## 4. Experimental Study

In this section, we compare CONNIA with three state-of-the-art MOEAs, including NNIA, NSGA-II, and SPEA2, on benchmark MOPs. Besides, some extensional problems based on the benchmark MOPs are also tested. It is well known that the parameter setting has significant impact on MOEAs. Therefore, the parameter setting of the four algorithms is consistent with the original references and has some adjustments appropriately. For SPEA2, the size of the population is 100; the size of an external population is 100. For NSGA-II, the size of the population is 100. For NNIA, the maximum size of the dominant population is 100; the maximum size of an active population is 20. For CONNIA, the maximum sizes of the two dominant subpopulations are both 50, and the maximum sizes of the two active subpopulations are both 10. A given number of function evaluations are used as the stopping criteria. We obtain statistical experimental results by running the four algorithms 30 times independently. To simplify the expression, Arabic numerals 1, 2, 3, and 4 are used to denote CONNIA, NNIA, NSGA-II, and SPEA2.

### 4.1. Evaluation Metrics

To evaluate various performances of the compared algorithms, some numerical metrics are adopted, including generation distance [44], spacing [45], maximum spread [36], hypervolume [19, 46], and the coverage of two sets [40]. These numerical metrics are summarized as follows.

*Generation Distance*. The metric which measures the distance from the approximate Pareto-optimal front to the true Pareto-optimal front is defined as follows:

(10) $$\begin{array}{c} \text{GD}\left(P,P^{*}\right)=\frac{\sum_{v\in P}d\left(v,P^{*}\right)}{\left|P\right|}, \end{array}$$

where P* is a set of uniformly distributed points in the objective space along the Pareto front, *P* is an approximation to the Pareto front, |*P*| is the number of solutions in *P*, and *d*(*v*, P*) is the minimum Euclidean distance between a point *v* in *P* and the solutions in P*.

*Spacing*. The metric measures the uniformity of nondominated solutions in the objective space and is described as follows:

(11) $$\begin{array}{c} S=\sqrt{\frac{1}{\left|A\right|-1}\sum_{i=1}^{\left|A\right|}{\left(\bar{d}-d_{i}\right)}^{2}}, \end{array}$$

where *d*_*i*_ = min⁡{∑_*m*=1_^*k*^|*f*_*m*_(*a*_*i*_) − *f*_*m*_(*a*_*j*_)|}, (*a*_*i*_, *a*_*j*_ ∈ *A*; *i*, *j* = 1,2,…, |*A*|), *d*_*i*_ is the distance between the solution *i* and another solution which is nearest to *i*, and $\bar{d}$ is the average value of all *d*_*i*_s.

*Maximum Spread*. The metric measures how “well” the true Pareto-optimal front is covered by the approximate Pareto-optimal front. It can be described as follows:

(12) $$\begin{array}{c} \text{MS}=\sqrt{\frac{1}{m}\sum_{i=1}^{m}{\left\{\frac{\min(f_{i}^{\max},F_{i}^{\max})-\max(f_{i}^{\min},F_{i}^{\min})}{F_{i}^{\max}-F_{i}^{\min}}\right\}}^{2},} \end{array}$$

where *m* is the number of objectives and *f*_*i*_^max⁡^ and *f*_*i*_^min⁡^ are the maximum and minimum values of the *i*th objective in the approximate Pareto-optimal front, respectively. *F*_*i*_^max⁡^ and *F*_*i*_^min⁡^ are the maximum and minimum values of the *i*th objective in the true Pareto-optimal front, respectively.

*Hypervolume*. The metric measures the “volume” in the objective domain covered by a set of nondominated solutions. The definition of the metric is

(13) $$\begin{array}{c} \text{HV}=\text{volume}\left(\overset{n_{PF}}{\underset{i=1}{⋃}}v_{i}\right), \end{array}$$

where *n*_*PF*_ is the number of nondominated solutions; for any nondominated solution *i*, a hypercube can be formed with a reference point and the solution *i* as the diagonal corners of the hypercube. Finally, the HV is the amount of domain occupied by the union of hypercubes.

*Coverage of Two Sets*. This metric measures the dominant relationship between two approximate Pareto-optimal sets *A* and *B*. The definition of the metric is described as follows:

(14) $$\begin{array}{c} I_{C}\left(A,B\right)≜\frac{\left|\left\{b\in B;\exists a\in A:a⪰b\right\}\right|}{\left|B\right|}, \end{array}$$

where the symbol “⪰” means domination. Note that both *I*_*C*_(*B*, *A*) and *I*_*C*_(*A*, *B*) have to be considered simultaneously, because the relationship between them is not completely linear.

### 4.2. Test Problems

To verify the versatility of the proposed algorithm, five ZDT [47] and five DTLZ problems [48] with diverse complexities in the field of multiobjective optimization are selected.  Table 1 demonstrates that NNIA may fall into local optimum in solving ZDT2 and ZDT4. So as to further explore the performance of CONNIA in solving some extreme problems, five test problems based on ZDT2 and ZDT4 are designed.

The related problems based on ZDT2 are described as follows. When *p* equals the values of 2 and 3, the corresponding problems are named ZDT21 and ZDT22, respectively. Consider the following:

(15) $$\begin{array}{c} g\left(x\right)=1+9{\left(\frac{\left(\sum_{i=2}^{n}x_{i}\right)}{\left(n-1\right)}\right)}^{0.25},\\ f_{1}\left(x\right)=x_{1},\;\;f_{2}\left(x\right)={\left\{g\left(x\right)\left[1-{\left(\frac{x_{1}}{g\left(x\right)}\right)}^{p}\right]\right\}}^{1/p}. \end{array}$$

**Figure 3:**
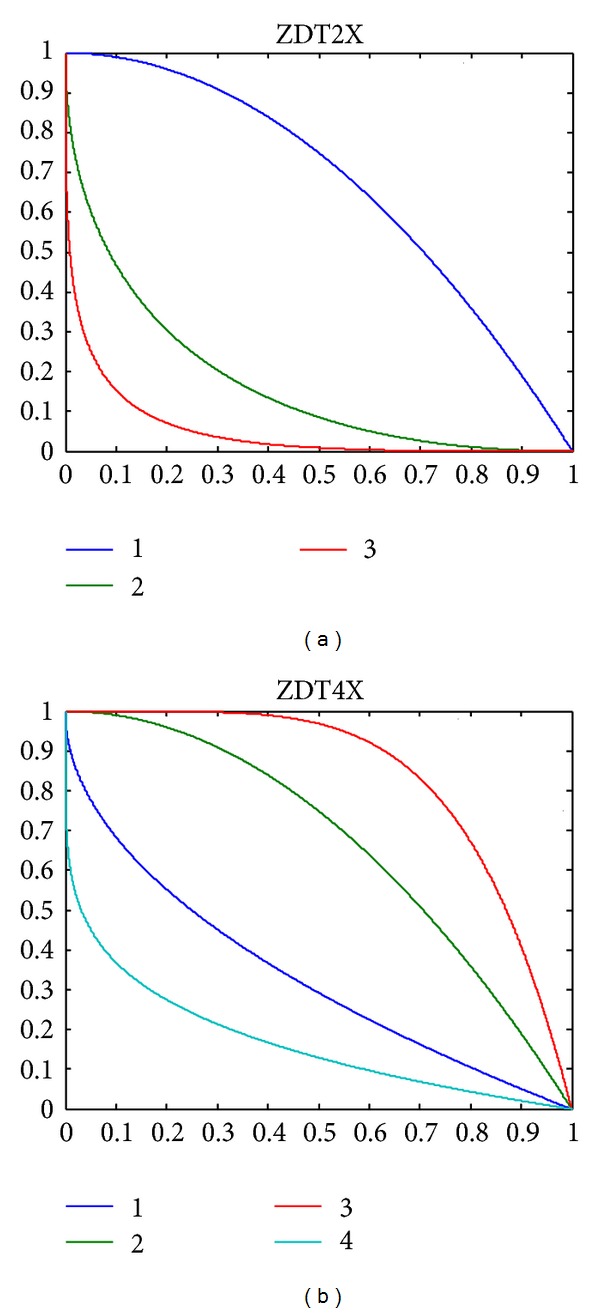
The true Pareto-optimal fronts of the related problems based on ZDT2 and ZDT4, respectively.

The shape of the Pareto-optimal front changes with the value of *p*. When *p* is greater than 1, the formative Pareto-optimal front is convex. If not, the formative Pareto-optimal front is concave. The curvature of the Pareto-optimal front also changes with the value of *p*. In Figure 3(a), we use Arabic numerals 1, 2, and 3 to concisely denote the Pareto-optimal fronts of ZDT2, ZDT21, and ZDT22, respectively.

Similar to the related problems based on ZDT2, the related problems based on ZDT4 are described as follows. When *q* equals the values of 0.5, 2, 5, and 0.2, the corresponding problems are named ZDT4, ZDT41, ZDT42, and ZDT43, respectively. Consider the following:

(16) f1(x)=x1, f2(x)=g(x)[1−(x1g(x))q], x1∈[0,1],g(x)=1+10(n−1)+∑i=2n[xi2−10cos⁡(4πxi)],    x∈[−5,5], i=2,…,n, n=10.

The shape of the Pareto-optimal front changes with the value of *q*. When *q* is greater than 1, the formative Pareto-optimal front is convex. If not, the formative Pareto-optimal front is concave. The curvature of the Pareto-optimal front also changes with the value of *q*. In Figure 3(b), we use Arabic numerals 1, 2, 3, and 4 to concisely denote the Pareto-optimal fronts of ZDT4, ZDT41, ZDT42, and ZDT43, respectively.

### 4.3. Sensitivity to the Introduced Parameter

**Figure 4:**
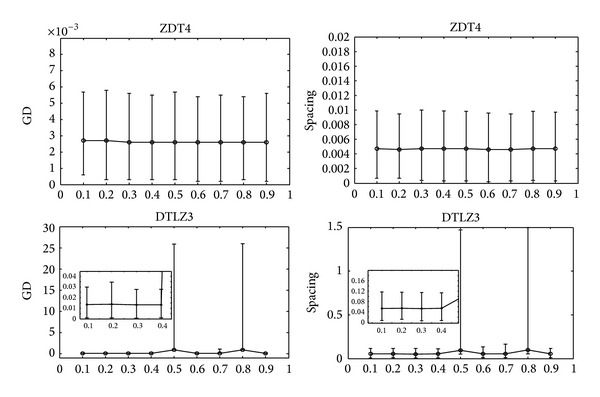
Mean values of GD and spacing versus the introduced parameter in solving DTLZ3 and ZDT4 by the proposed algorithm.

The influence of the threshold is discussed in this part. Considering the representative of multi-objective problems with two-objectives and three-objectives, respectively, ZDT4 and DTLZ3 are selected for parameter analysis.  Figure 4 shows that the mean values of GD and spacing are rather stable in dealing with ZDT4, whatever the value of the threshold is. However, the mean values of GD and spacing change greatly with the variation of the threshold in solving DTLZ3.  Figure 4 indicates that the proposed algorithm is not sensitive to the threshold on simple problems. The performance has some differences with the variation of the threshold on difficult problems. When the cooperation among subpopulations happens with a small value of the threshold, the information among subpopulations will keep coincidence with each other which leads to the ineffectiveness of the cooperation. On the contrary, when the late cooperation appears with a large value of the threshold, the differences among subpopulations are apparent. Thus, there is little chance for the inferior subpopulation to gain experience from the superior one.

### 4.4. Comparison of CONNIA with and without Information Exchange

The cooperation operator reflects a mutually beneficial relationship between two subpopulations. By applying the cooperation operator, two subpopulations gain the opportunity to exchange information and expand the search range of the entire population. The subpopulation could make use of the reference experience from each other to improve its own evolution. This directed cooperation operator provides good evolutionary paths towards antibodies, thereby making antibodies evolve faster.

**Figure 5:**
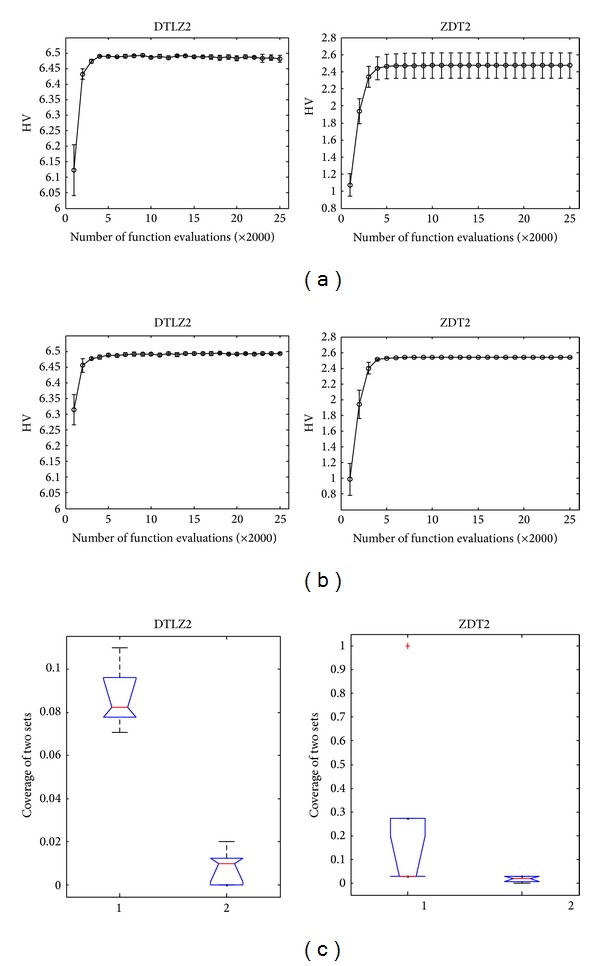
(a) The error bar of HV of the nondominated antibodies in final population with different number of function evaluations by CONNIA′. (b) The error bar of HV of the nondominated antibodies in final population with different number of function evaluations by CONNIA. (c) Box plots of the coverage of the two sets obtained by CONNIA with and without information exchange.

In this part, the effectiveness of information exchange among subpopulations is discussed. The proposed algorithm without information exchange is denoted by CONNIA′. Figures 5(a) and 5(b) show the error bars of hypervolume metric of nondominated antibodies in final population with different number of function evaluations by CONNIA and CONNIA′, respectively. From Figures 5(a) and 5(b), some conclusions can be obtained: (1) the evolution curves of CONNIA are more flat than those of CONNIA′; (2) the standard deviation of the error bar obtained by CONNIA becomes near to zeros; (3) with the same number of function evaluations, CONNIA obtains a higher value of HV metric than CONNIA′.  Figure 5(c) shows the box plots of CONNIA against CONNIA′ in terms of the coverage of two sets. In each plot, the left box represents the distribution of *I*_*C*_(CONNIA, CONNIA′) and the right box represents the distribution of *I*_*C*_(CONNIA′, CONNIA). The box plots of *I*_*C*_(CONNIA, CONNIA′) are higher than the corresponding box plots of *I*_*C*_(CONNIA′, CONNIA). Therefore, we can get the conclusion that CONNIA performs better than CONNIA′ as far as the coverage is concerned.

### 4.5. Experimental Results on ZDT and DTLZ Problems

**Figure 6:**
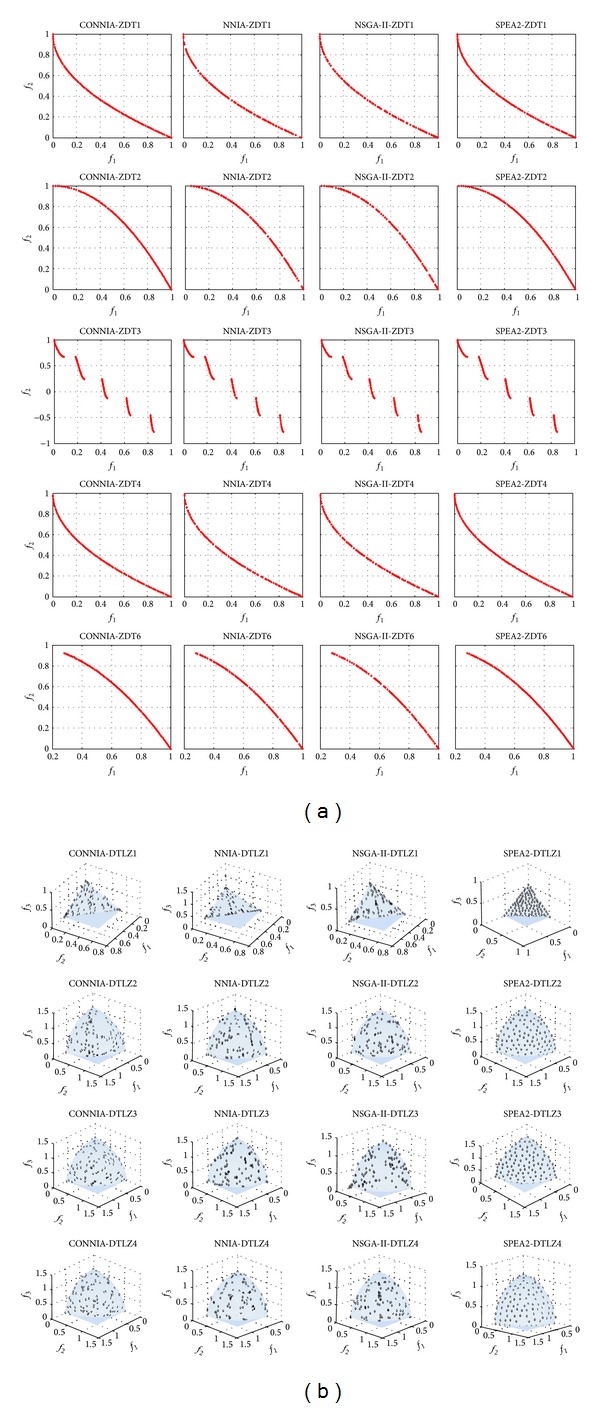
The approximate Pareto-optimal fronts obtained by CONNIA, NNIA, NSGA-II, and SPEA2 in solving the 9 test problems.

**Figure 7:**
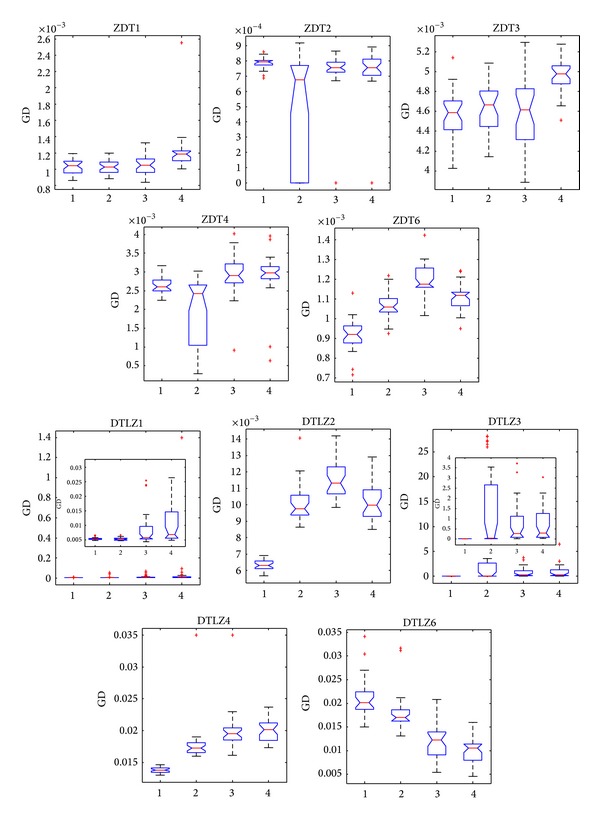
Statistical values of convergence obtained by CONNI, NNIA, NSGA-II, and SPEA2 in solving the 10 test problems.

**Figure 8:**
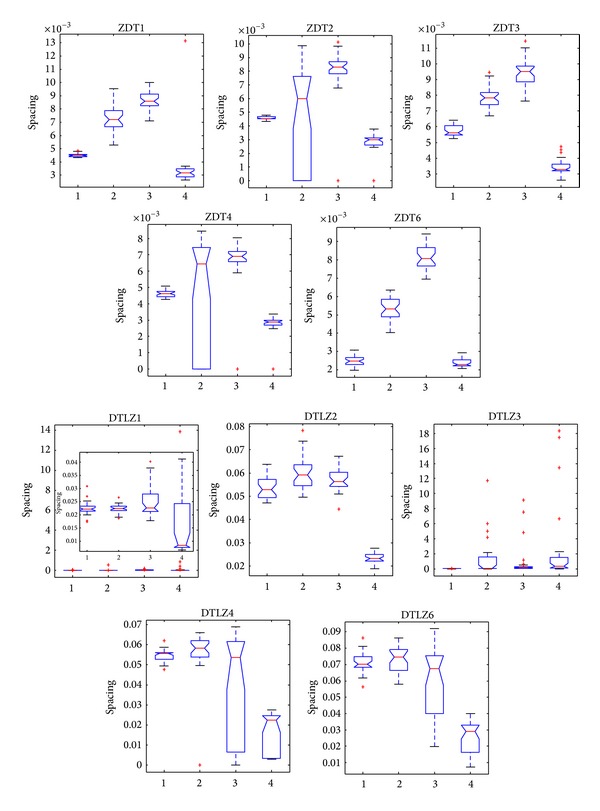
Statistical values of spacing obtained by CONNI, NNIA, NSGA-II, and SPEA2 in solving the 10 test problems.

**Figure 9:**
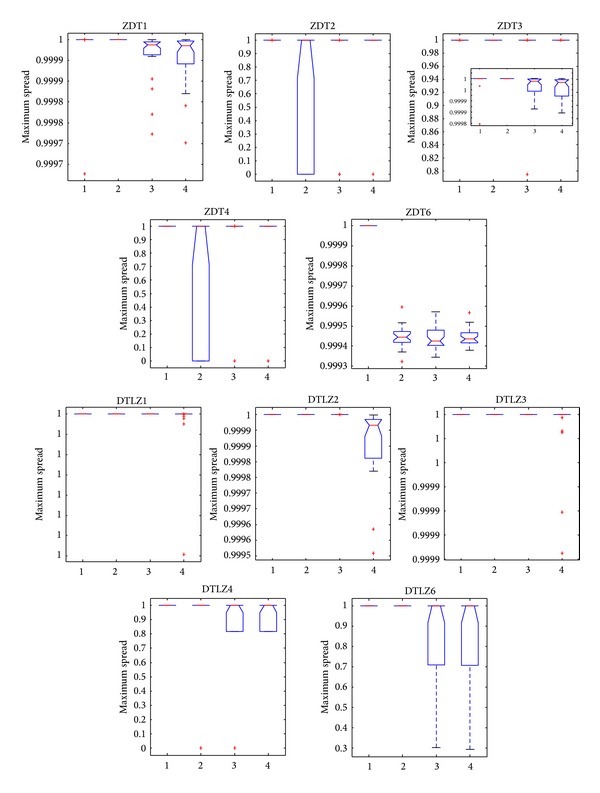
Statistical values of maximum spread obtained by CONNI, NNIA, NSGA-II, and SPEA2 in solving the 10 test problems.

**Figure 10:**
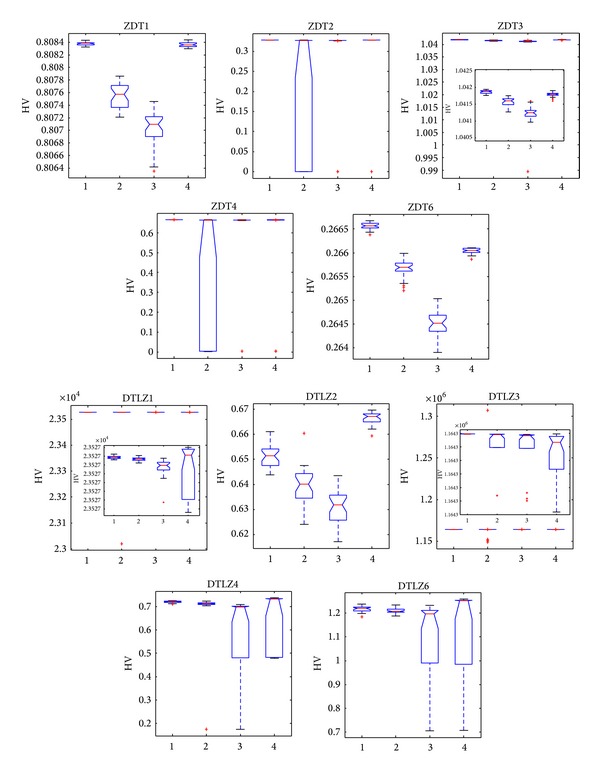
Statistical values of HV obtained by CONNI, NNIA, NSGA-II, and SPEA2 in solving the 10 test problems.

Figure 6 shows the distribution of approximate Pareto-optimal solutions obtained by four algorithms on ZDT and DTLZ problems. The distributions of the approximate Pareto-optimal solutions obtained by CONNIA and SPEA2 are more uniform than those obtained by other two algorithms on five ZDT problems. The approximate Pareto-optimal solutions obtained by NNIA can not well cover the extreme solutions of ZDT2 and ZDT4. For DTLZ problems, the distribution of the approximate Pareto-optimal solutions obtained by SPEA2 is the most uniform among the four algorithms; nevertheless the computational complexity of SPEA2 is the highest. The distribution of the approximate Pareto-optimal solutions obtained by CONNIA is the most uniform among the remaining three algorithms. In addition to the qualitative analysis of the results, we also analyze statistical results obtained by four algorithms. The statistical results of convergence, spacing, maximum spread, and hypervolume are shown in Figures 7–10.

Figure 7 shows that the values of convergence can reach 10^−3^ in almost all the 30 independent runs by four algorithms on five ZDT problems. The box plots obtained by NNIA on ZDT2 and ZDT4 are quite broad which indicates that the stability of NNIA in solving these problems is quite poor. However, CONNIA is more robust than NNIA on ZDT2 and ZDT4, owing to the multipopulation coevolutionary strategy which plays an important role in maintaining the population diversity. In general, except for the appearance of local optimum when NNIA deals with ZDT2 and ZDT4, the differences among four algorithms on five ZDT problems are relatively small. Hereinto, CONNIA obtains the smallest values of convergence on ZDT3, ZDT4, and ZDT6. It has been pointed out that NSGA-II and SPEA2 could not completely converge onto the true Pareto-optimal fronts in a limited number of function evaluations on DTLZ3 which has some local Pareto-optimal fronts [35, 49]. However, CONNIA obtains the best results in terms of convergence on DTLZ3. As far as convergence is concerned, CONNIA performs best on DTLZ1, DTLZ2, DTLZ3, and DTLZ4.

Figure 8 shows that, compared with the other three algorithms, SPEA2 performs best in most problems in terms of spacing. Apart from SPEA2, statistical values obtained by CONNIA are smaller than those obtained by other two algorithms in 9 out of the 10 problems. The statistical value obtained by CONNIA is even smaller than that obtained by SPEA2 on DTLZ3. The reason is that SPEA2 can not quite converge onto the true Pareto-optimal fronts in a limited number of function evaluations. In general, SPEA2 exhibits the best performance in diversity maintaining among the four algorithms. However, the complicated calculation of SPEA2 costs a large amount of computing resources. The proposed algorithm gets the smallest values of spacing among the remaining three algorithms in solving ZDT1, ZDT2, ZDT3, ZDT4, ZDT6, DTLZ1, DTLZ2, DTLZ3, and DTLZ4.

Figure 9 demonstrates that NNIA obtains broad box plots on ZDT2 and ZDT4, thereby suggesting that the stability of NNIA on ZDT2 and ZDT4 is poor. Compared with the other three algorithms, CONNIA obtains the largest statistical values of MS on all the 10 test problems, while NSGA-II and SPEA2 perform slightly poor on ZDT1, ZDT3, ZDT6, DTLZ4, and DTLZ6.  Figure 10 shows that the stability of NNIA is quite poor on ZDT2 and ZDT4 in terms of HV. However, in most of the 10 test problems except DTLZ2, the result obtained by the CONNIA is not inferior to that obtained by other three algorithms as far as HV is concerned. Apparently, SPEA2 does well in diversity maintenance in the field of MOEAs. In solving five ZDT problems, SPEA2 gets the results similar to CONNIA in terms of HV. However, SPEA2 can not well converge onto the true Pareto-optimal fronts in 50000 function evaluations in solving difficult problems. CONNIA achieves the results which are not worse than, or even better than, those of SPEA2 with much lower complexity on the nine test problems.

### 4.6. Comparing the Robustness of NNIA and CONNIA

**Figure 11:**
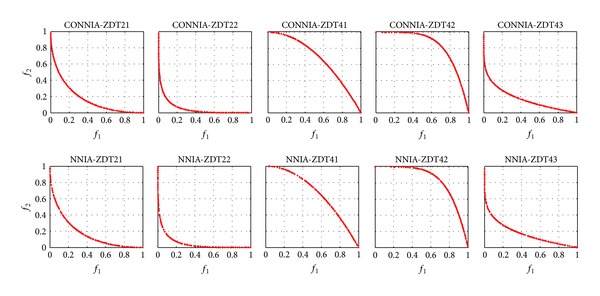
The approximate Pareto-optimal fronts obtained by CONNIA and NNIA in solving the extensional problems.

The comparison of CONNIA and NNIA on some difficult problems (DTLZ1 and DTLZ3) and some extreme problems (ZDT21, ZDT22, ZDT41, ZDT42, and ZDT43) is carried out in this part.  Figure 11 shows the distribution of approximate Pareto-optimal solutions obtained by CONNIA and NNIA. The distributions of approximate Pareto-optimal solutions obtained by CONNIA are relatively more uniform than those of NNIA. The solutions obtained by NNIA can not well cover extreme solutions in solving ZDT21, ZDT41, and ZDT42. Nevertheless, CONNIA can well cover these solutions in solving the same problems.

**Figure 12:**
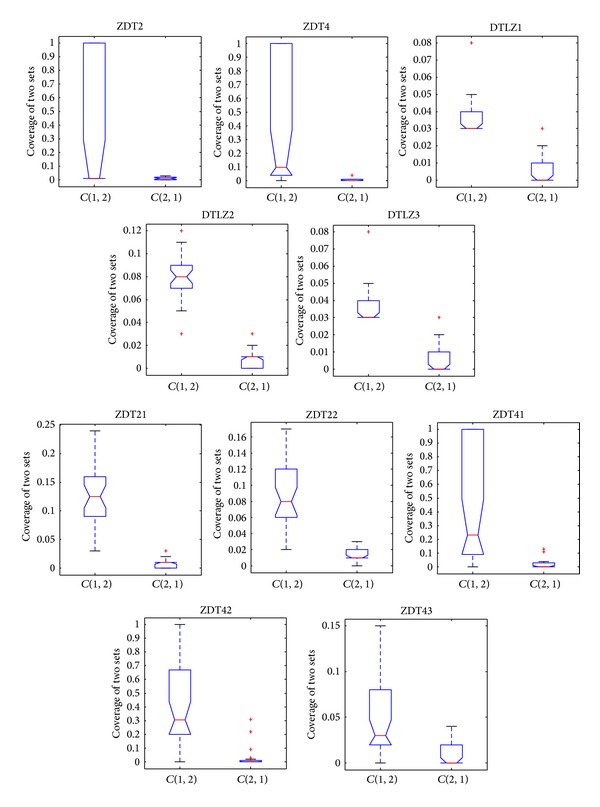
Box plots of the coverage of two sets by CONNIA and NNIA in solving 10 test problems.

Figure 12 shows the box plots of CONNIA against NNIA based on the coverage of two sets. NNIA obtains a relatively wider range of box plot measures on ZDT2, ZDT4, and ZDT41; that is, the stability of NNIA is relatively weak in dealing with these problems. However, the performance obtained by CONNIA is more stable on the same problems. The box plots of *C*(1,2) are higher than the corresponding box plots of *C*(2,1) in all the test problems as shown in Figure 12. Therefore, we can get the conclusion that the solutions obtained by CONNIA almost weakly dominate those obtained by NNIA.

### 4.7. Tests on Convergence of the Four Algorithms

In the field of MOEAs, the number of function evaluations is commonly used as the stopping criteria. It is difficult to set the accurate stopping criteria for an MOEA on different problems, while uniform stopping criteria which are applied to different problems always provide a plethora of information [49]. After investigating the running convergence with different function evaluations, the effective stopping criteria of CONNIA on different problems can be discovered. To demonstrate the convergence of four algorithms more explicitly, results are showed with *Y* coordinate in the form of log 10.

**Figure 13:**
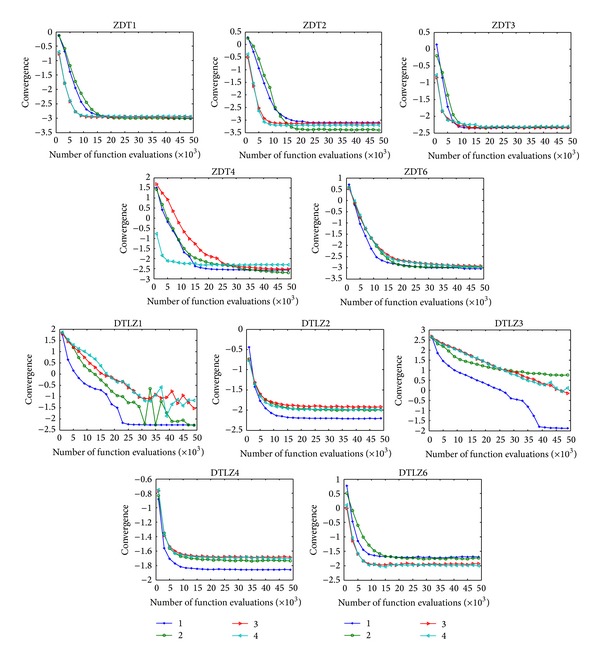
Mean value of convergence with different function evaluations by the four algorithms in solving the 10 test problems.

Figure 13 shows the mean value in terms of convergence with different function evaluations by four algorithms. The differences among four algorithms are not obvious on five ZDT problems. However, the disparities among them are apparent on five DTLZ problems. CONNIA obtains better performance than the other three algorithms on DTLZ1, DTLZ2, DTLZ3, and DTLZ4. In particular on some intractable problems, such as DTLZ1 and DTLZ3, SPEA2 and NSGA-II can not well converge onto the true Pareto-optimal front with a limited number of function evaluations, while under the same condition CONNIA shows distinct advantages.

### 4.8. Experimental Results of the Four Algorithms on Many-Objective Problems

In this section, the performance of four algorithms on many-objective problems is investigated. Multiobjective problems with more than three objectives are defined as many-objective problems. The test problems are the extensional problems of DTLZ1 and DTLZ2 with 4 to 7 objectives and are named DTLZ14–DTLZ17 and DTLZ24–DTLZ27, respectively. Due to the fact that the number of nondominated solutions dramatically enlarges with the number of objectives increasing, many MOEAs have difficulty in converging onto the true Pareto-optimal front with a limited number of function evaluations. Therefore, the size of population and the number of function evaluations are doubled as those in Section 4.5 [35].

**Figure 14:**
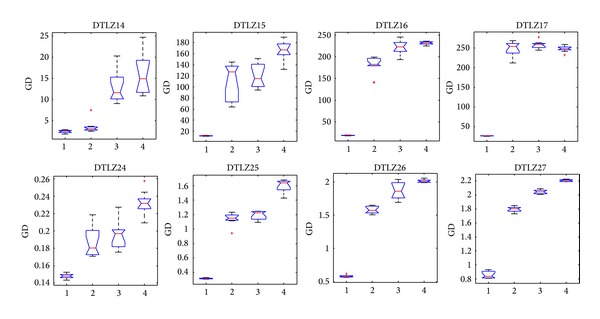
Statistical values of convergence obtained by the four algorithms in solving many-objective problems.

**Figure 15:**
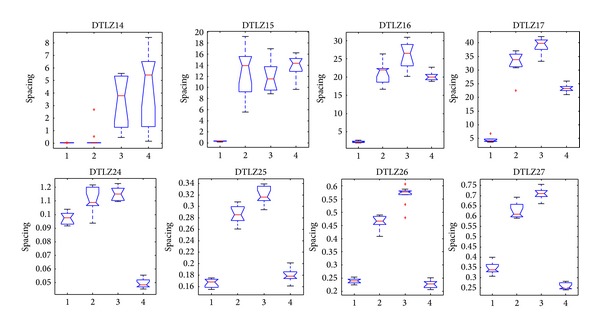
Statistical values of spacing obtained by the four algorithms in solving many-objective problems.

**Figure 16:**
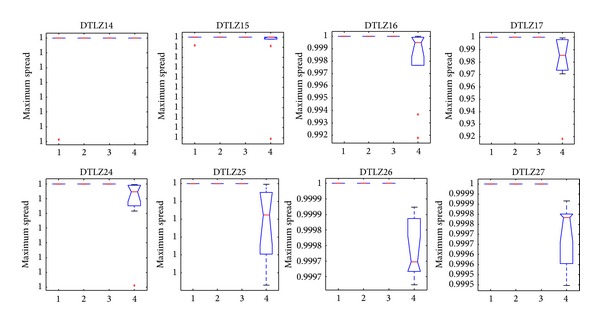
Statistical values of maximum spread obtained by the four algorithms in solving many-objective problems.

Figure 14 shows that CONNIA obtains the largest statistical values of convergence among four algorithms on all the test problems, closely followed by NNIA. While results obtained by SPEA2 and NSGA-II are relatively worse in terms of convergence, Figure 15 indicates that the result of CONNIA is even better than SPEA2 in terms of spacing. SPEA2 cannot converge onto the true Pareto-optimal fronts with a limited number of function evaluations on eight many-objective problems. In this case, the diversity maintaining mechanism used in SPEA2 is no longer effective. The statistical values of MS on eight many-objective problems are shown in Figure 16. In terms of MS, four algorithms obtain similar results, except SPEA2 which does slightly worse. Overall, CONNIA performs much better than the other three algorithms on eight many-objective problems. The performance of NSGA-II and SPEA2 is seriously degenerated in solving many-objective problems.

**Figure 17:**
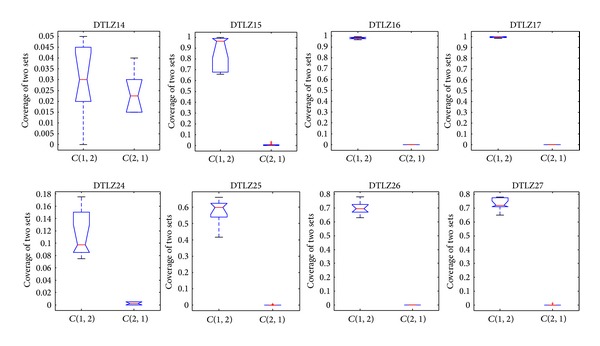
Statistical values of the coverage of two sets obtained by CONNIA and NNIA in solving many-objective problems.

**Figure 18:**
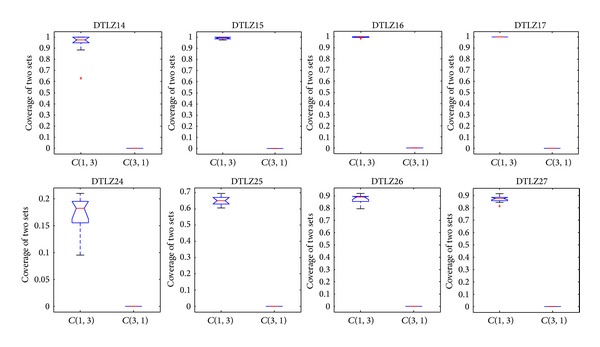
Statistical values of the coverage of two sets obtained by CONNIA and NSGA-II in solving many-objective problems.

**Figure 19:**
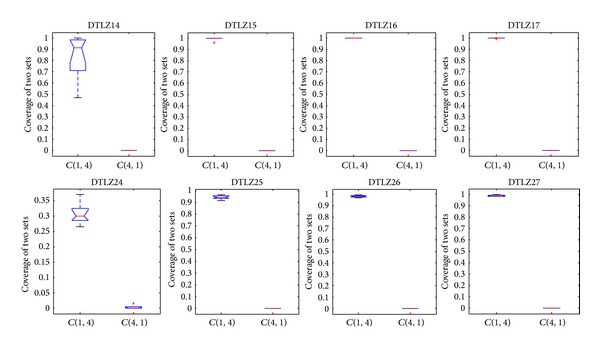
Statistical values of the coverage of two sets obtained by CONNIA and SPEA2 in solving many-objective problems.

The convergence metric can be only used under the condition of which knowledge of the true Pareto-optimal fronts is available, which is unsuitable for many-objective problems. Hence, the metric of the coverage of two sets is employed to measure the dominant relationship between solutions obtained by different algorithms. Figures 17, 18, and 19 show the comparison between CONNIA and the other three algorithms on many-objective problems in terms of the coverage of two sets. Figures 17–19 indicate that the values of *C*(∗, 1) are smaller than the corresponding values of *C*(1, ∗). Hereinto, 1 denotes the solution set obtained by CONNIA, and the symbol “∗” stands for the solution set obtained by any one of the other three algorithms. The gap between *C*(∗, 1) and *C*(1, ∗) is enlarged with the number of objectives increasing, which indicates that the dominant relationship between the solutions obtained by CONNIA and other three algorithms is more apparent on complex many-objective problems. In the special case that *C*(1, ∗) = 1 and *C*(∗, 1) = 0, the solution set obtained by CONNIA almost dominates that obtained by any one of the other three algorithms. For example, in solving DTLZ15, DTLZ16, DTLZ17, and DTLZ27, the values of *C*(∗, 1) are almost equal to 0, while the values of *C*(1, ∗) are almost equal to 1. CONNIA outperforms the other three algorithms in most cases as coverage is concerned.

### 4.9. Running Time Study

**Figure 20:**
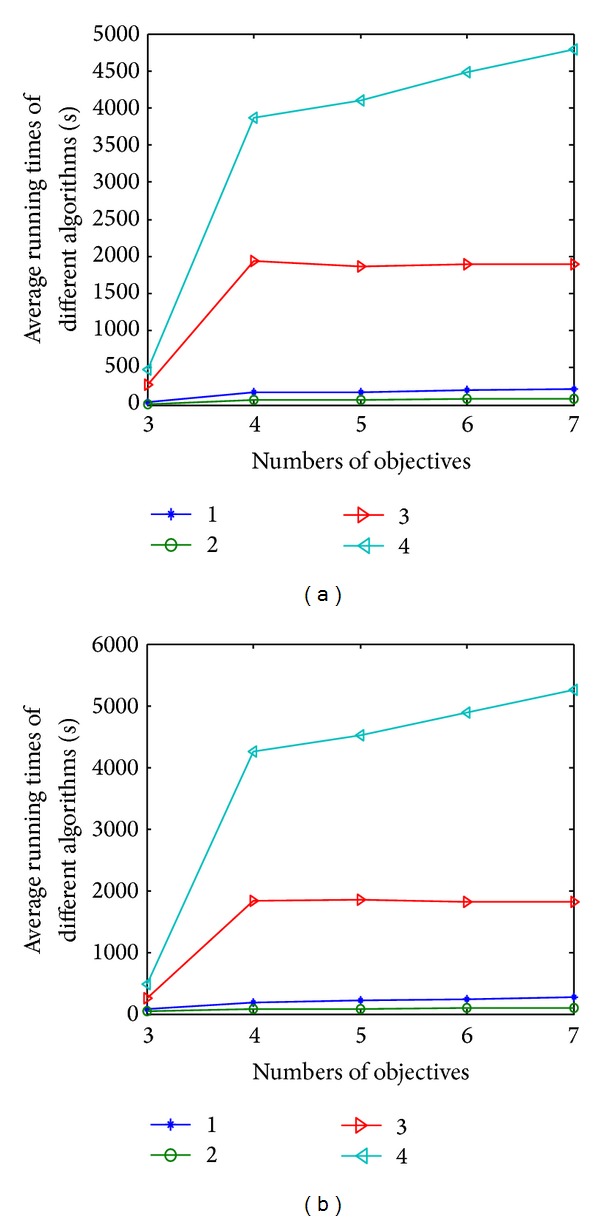
Mean value of running time by four algorithms on the extensional problems of DTLZ1 and DTLZ2 with 3 to 7 objectives, respectively.

Figure 20 shows the average running time on the extensional problems of DTLZ1 and DTLZ2 with 3 to 7 objectives, respectively. As shown in Figure 20, the cost of the average running time of four algorithms increases with the number of objectives increasing. NNIA exhibits the best performance in terms of computational time, closely followed by CONNIA. The running time of NSGA-II and SPEA2 is relatively longer; particularly for SPEA2, the required running time is the longest among the four algorithms. This is because SPEA2 adopts a relatively expensive diversity maintaining mechanism whose worst run-time complexity is *O*(*N* ^3^), where *N* is the number of nondominated solutions. NNIA is an effective immune inspired MOEA, which is famous for good performance in convergence [35, 50]. Although its special evolutionary framework results in fast convergence, solutions obtained by NNIA are occasionally trapped into local optimum. It is required to focus on the pursuit of not only a high convergence rate, but also good evolutionary quality. CONNIA is an enhanced version of NNIA by introducing the multipopulation coevolutionary strategy and an adaptive operator. Although the computational cost of CONNIA is a little larger than NNIA, the improvement on the performance is evident.

## 5. Conclusion 

To the best of our knowledge, slow convergence rate is a ubiquitous problem in MOEAs. AIS have the learnable, parallel, and distributed characteristics and possess an efficient information processing ability. AIS-based algorithms have already been widely used for dealing with MOPs, in which NNIA obtains a fast convergence rate solving such knotty problem in MOEAs. However, the population diversity can not be well maintained in NNIA, which leads the solutions obtained by NNIA to be trapped into local optimum on some difficult problems. Co-evolution is a high-level evolutionary method, which confirms that all the populations are beneficial mutually, thus providing a theoretical basis for maintaining diversity. In this paper, a multipopulation coevolutionary strategy is designed. Subpopulations implement evolutionary operation independently; thus the diversity of the entire population can be well maintained. The information exchange among subpopulations is available, thereby expanding the search range of the entire population and improving the efficiency of evolutionary search.

In the field of MOEAs, when it comes to adaptive algorithms, most of them adaptively adjust some parameters, such as population size, crossover probability, and mutation probability. However, an adaptive algorithm with online-decision strategy is seldom involved. Based on this idea, an adaptive strategy is designed in the proposed algorithm. Subpopulations adopt corresponding operations according to the conditions of themselves which ensures that evolutionary search is not random or blind.

In dealing with many-objective problems, the rapid increase of nondominated solutions requires a large size of population or a large number of function evaluations. However, in many MOEAs, the size of population is constant. No matter how difficult the problem is, the size of population is the same. According to the characteristics of CONNIA, it is more reasonable to adaptively adjust the number of subpopulations according to the difficulty of the problem. We can imagine that it is more reasonable to employ more subpopulations together to cooperatively overcome the difficulty in solving many-objective problems.

## Figures and Tables

**Table 1:** The situation of falling into local optimum.

Test problems	ZDT2	ZDT4
Times of falling into local optimum	12 times	10 times
The number of nondominated solutions	1	1
